# A proposed clinical and biological interpretation of mediated interaction

**DOI:** 10.1007/s10654-015-0087-5

**Published:** 2015-10-05

**Authors:** M. Arfan Ikram, Tyler J. VanderWeele

**Affiliations:** Departments of Epidemiology, Neurology, and Radiology, Erasmus University Medical Center, PO Box 2040, 3000 CA Rotterdam, The Netherlands; Departments of Epidemiology and Biostatistics, Harvard TH Chan School of Public Health, Boston, MA USA

**Keywords:** Mediation, Mediated interaction, Four-way decomposition, Causal inference, Total effect, Indirect effect, Direct effect, Clinical medicine

## Abstract

Understanding of causal pathways in epidemiology involves the concepts of direct and indirect effects. Recently, causal mediation analysis has been formalized to quantify these direct and indirect effects in the presence of exposure–mediator interaction and even allows for four-way decomposition of the total effect: controlled direct effect, reference interaction, mediated interaction, pure indirect effect. Whereas the other three effects can be intuitively conceptualized, mediated interaction is often considered a nuisance in statistical analysis. In this paper, we focus on mediated interaction and contrast it against pure mediation. We also propose a clinical and biological interpretation of mediated interaction using three hypothetical examples. With these examples we aim to make researchers aware that mediated interaction can actually provide important clinical and biological information.

## Background

Recently, the conceptual framework of causal mediation has been formalized to allow quantitative analysis of mediation, i.e. the estimation of indirect and direct effects. The indirect effect refers to the effect that is through the mediator under study. The direct effect refers to the remaining effect that is not through the mediator. The causal inference literature on mediation now allows for the estimation of these effects even in the presence of exposure–mediator interaction [1]. More recently, a four-way decomposition of the total effect (*TE*) has been suggested [2], in which the direct and indirect effects are further subdivided into four components. These four effects are referred to as the controlled direct effect (*CDE*), reference interaction effect (*INT*_*ref*_), mediated interaction effect (*INT*_*med*_); and pure mediated (or indirect) effect (*PIE*).

In this paper we consider mediated interaction, which might be seen as intuitively more difficult, and so far lacks an easy-to-understand clinical or biological interpretation. We do so by describing three hypothetical examples of mediated interaction, which, in these examples, is recognized more easily by its clinical or biological terminology. We start by briefly discussing the theoretical background of counterfactuals. We then explore the theoretical difference between mediated interaction and pure mediation, since these effects seem very similar to each other but in fact are distinct entities. Finally, we describe the three examples and highlight the clinical or biological phenomenon that reflects mediated interaction in each of these scenarios (Table 1). The reader is advised that we do not aim to develop new methodology, but instead only use existing theoretical concepts and illustrate these using easily interpretable examples.

**Table 1:** Clinical and biological examples of mediated interaction

	Exposure (*A*)	Mediator (*M*)	Outcome (Y)
Example 1	Risk factor for reversible damage	Reversible damage	Disease
Example 2	Unstable proteins in the brain	First misfolded protein	Creutzfeldt-Jacob’s disease
Example 3	Group of male clownfish	Female clownfish	Offspring

## Counterfactuals

An important cornerstone in causal mediation analysis is the theoretical framework of counterfactuals or potential outcomes. Here, we provide a brief summary of the counterfactual notation and its application in causal mediation analysis [1, 2]. In the current report we follow most of the rest of the literature and consider notations for counterfactuals that pertain to a single moment in time for each of the exposure, mediator, and outcome. Currently, methods to generalize these concepts to incorporate time-varying exposures and mediators are being developed [3].

Consider a context in which mediation between an exposure and outcome may be present [1, 2], and let *A* by an exposure, *M* a mediator and *Y* an outcome, each measured at specified time points. Throughout this paper, for simplicity, we will restrict our attention to the case in which *A*, *M* and *Y* are all binary. Additionally, for simplicity, we assume that the positive monotonicity assumption holds, that the exposure does not prevent the mediator or outcome and also that the mediator does not prevent the outcome for any individual [1]. Within the counterfactual framework, for each individual in a considered population we may define *Y*_*a*_ as the potential outcome *Y* we would have observed if *A* had been set to *a*. The total effect (*TE*) of *A* on *Y* is then given by *Y*_1_ − *Y*_0_. We define *Y*_*am*_ as the potential outcome *Y* if, possibly contrary to fact, *A* were set to *a* and *M* were set to *m*. Similarly we may define *M*_*a*_ as the potential mediator *M* if, possibly contrary to fact, *A* were set to *a*. We make a composition assumption that (*Y*_*a*_ = *Y*_*aMa*_).

The four-way decomposition of the total effect can be written as:

$$ TE = Y_{1} {-}Y_{0} = CDE + INT_{ref} + INT_{med} + PIE $$

In this expression, the four components are as follows:

The controlled direct effect (*CDE*) is given by (*Y*_10_ − *Y*_00_) and is the component due to neither mediation nor interaction, i.e. the effect of the exposure on outcome in the absence of the mediator.

The reference interaction (*INT*_*ref*_) is given by (*Y*_11_ − *Y*_10_ − *Y*_01_ + *Y*_00_) *M*_0_ and is the component due to interaction but not mediation, i.e. the interactive effect when the mediator is left to the level it would take in absence of exposure.

The mediated interaction (*INT*_*med*_) is given by (*Y*_11_ − *Y*_10_ − *Y*_01_ + *Y*_00_) (*M*_1_ − *M*_0_) and is the component due both mediation and interaction.

The pure indirect effect (*PIE*) is given by (*Y*_01_ − *Y*_00_) (*M*_1_ − *M*_0_) and is the component due to just mediation, not interaction.

## Mediated interaction versus pure mediation

In order to aid the interpretation of mediated interaction and its contrast with pure mediation, we make use of two hypothetical scenarios: one in which the total effect of exposure on outcome is entirely due to mediated interaction and one in which the total effect is entirely due to pure mediation. From the counterfactual framework these scenarios are defined as:

$$ \begin{array}{*{20}l} {{\text{Scenario }}1:TE = INT_{med} = \left( {Y_{11} {-}Y_{10} {-}Y_{01} + Y_{00} } \right)(M_{1} {-}M_{0} );{\text{ and}}} \hfill \\ {{\text{Scenario }}2:TE = PIE = \left( {Y_{01} {-}Y_{00} } \right)(M_{1} {-}M_{0} )} \hfill \\ \end{array} $$

Under monotonicity, these will be non-zero only if at least the following three conditions are met:

- *M*_0_ = 0 to rule out any reference interaction so that in absence of exposure the mediator is also absent; and
- (*Y*_10_ − *Y*_00_) = 0 to rule out any pure direct effect so that the exposure has no effect on outcome in absence of mediator; and
- (*M*_1_ *−* *M*_0_) ≠ 0 indicating that the exposure has an effect on the mediator, i.e. given condition 1 results in *M*_1_ ≠ 0.

The difference then between scenario 1 and 2 for the mediated interaction and the pure indirect effect lies in the first term of the two products. Importantly, the first term in the product of scenario 1 can be re-written as (*Y*_11_ − *Y*_10_) − (*Y*_01_ − *Y*_00_). This compares to the first term in the product of scenario 2, which is (*Y*_01_ − *Y*_00_).

Under monotonicity, for mediated interaction as in scenario 1, it is required that (*Y*_01_ − *Y*_00_) = 0 to rule out any *PIE*, while requiring (*Y*_11_ − *Y*_10_) ≠ 0. For pure mediation as in scenario 2, it is required that (*Y*_01_ − *Y*_00_) ≠ 0 and that (*Y*_11_ − *Y*_10_) = (*Y*_01_ − *Y*_00_), which given condition 2 reduces to *Y*_11_ = *Y*_01_ or alternatively (*Y*_11_ − *Y*_01_) = 0.

In other words, in addition to the three conditions described above for mediated interaction it is required that the mediator has an effect on the outcome only in the presence of the exposure, but not in the absence of exposure, i.e. the exposure is necessarily present for the mediator to affect outcome. For pure mediation, in addition to the three conditions described above it is required that the mediator has an effect on the outcome in the absence of the exposure.

## Example 1: reversible versus irreversible damage

The first example will illustrate the clinical difference between mediated interaction and pure mediation and involves the concepts of reversible and irreversible damage [4]. There are many diseases (*Y*) for which a risk factor (*A*) leads to reversible damage (*M*), i.e. (*M*_1_ − *M*_0_) ≠ 0. If the risk factor was not present, such reversible damage regresses and therefore cannot lead to disease [i.e. (*Y*_01_ − *Y*_00_) = 0]; however, if the risk factor is present such reversible damage can result in clinical disease [i.e. (*Y*_11_ − *Y*_10_) ≠ 0]. This is analogous to scenario 1 and represents mediated interaction, since the exposure must be present for the mediator to affect the outcome. An example is gastric acid reflux (*A*), reflux esophagitis (*M*) and esophagus cancer (*Y*). There are other diseases, for which a risk factor (*A*) leads to irreversible damage (*M*). In these instances however, even if the risk factor is not present, the damage is irreversible and can go on to manifest itself as clinical disease [i.e. (*Y*_01_ − *Y*_00_) ≠ 0]. This is analogous to scenario 2 and represents pure mediation, since the exposure no longer needs to be present for the mediator to affect the outcome. An example is high radiation exposure (*A*), gonadal damage (*M*) and infertility (*Y*). In reality, for many diseases, mediated interaction and pure mediation will co-occur. Alternatively, at low levels of exposure the effect might be mediated interaction (reversible damage), whilst at high exposure level the effect may become pure mediation (irreversible damage). Nevertheless, we feel that these examples, though a simplification of actual disease processes, may serve as clinical illustrations of mediated interaction and pure mediation.

## Example 2: auto-catalysis

The second example originates from biochemical sciences, in which mediated interaction is perhaps best illustrated by a process called auto-catalysis. This is a process in which a certain chemical reaction only takes place after a certain small amount of intermediate has been formed. This small amount of intermediate then acts as trigger to dramatically increase the speed (catalyze) of the reaction [5]. In other words, the exposure causes the mediator and the mediator interacts with exposure to cause outcome. Auto-catalytic processes have been described in DNA replication and the spontaneous degradation of aspirin into salicylic acid and acetic acid, among others. Interestingly, autocatalysis has even been considered an instance of positive feedback [5]. In clinical medicine, prion diseases, such as Creutzfeldt-Jacob’s disease, involve auto-catalysis and might serve to demonstrate mediated interaction. Prion diseases are caused by the abrupt and quick accumulation of misfolded prion proteins in the brain leading the neuronal cell death, dementia and ultimately death [4]. The pathologic mechanism is thought to involve the misfolding of an initial single protein, for instance due to external inoculation of misfolded protein or a genetic variant leading to unstable normal proteins more prone to misfolding. It is then an initial misfolded protein that acts as template for other proteins to become misfolded as well, thereby triggering a chain reaction leading to rapid accumulation of misfolded proteins, which then manifests itself as a rapidly progressing dementia syndrome. If we focus for instance on the pathway that involves a genetic variant, unstable proteins, the first unstable protein to misfold, exponential misfolding of other proteins and ultimately cell-death and dementia, it involves a certain degree of mediated interaction. The causal pathway from unstable proteins (*A*) to dementia (*Y*) goes entirely via the first misfolded protein (*M*). However, the first misfolded protein (*M*) in itself will not cause dementia, since it is requires other unstable proteins (*A*) to be present, which it triggers to become misfolded as well. Of course, there are evident differences between biochemically autocatalytic processes and prion disease. For instance, while some biochemical processes may entirely depend on autocatalysis, in prion diseases although the first misfolded protein will not in itself give rise to full-blown dementia without the presence of other proteins to be misfolded, it might in itself lead to certain small amount of cell death (pure mediation) or alternatively, external inoculation of the first misfolded protein might trigger the exponential cascade leading to cell-death (reference interaction). Nevertheless, the parallels between mediated interaction and autocatalysis are striking and may aid in further conceptualizing its interpretation.

## Example 3: gender-change and offspring in clownfish

Finally, we use animal biology for an exotic example of mediated interaction. A certain species of fish, called *Amphiprioninae* or clownfish, have an intriguing life-cycle, which involves changing of gender [6]. Clownfish are all born male and, within a group of clownfish, only one adult male undergoes a gender change to become female. This female lays eggs that are fertilized by an adult male to produce offspring. The lifecycle of clownfish is an example of mediated interaction: it involves a group of young male clownfish (*A*), and the gender change of a single fish to female (*M*), while at the same time the female only produces offspring (*Y*) after mating with a male (*A*). The group of male clownfish produces the single female fish, but both the group of male clownfish and the female clownfish are needed for the offspring. We also note that males cannot produce offspring without females (i.e. there is no direct effect), females cannot produce offspring without males (i.e. there is no pure mediated effect), and females cannot exist without first being a male (i.e. there is no reference interaction).

## Conclusion

Causal mediation analysis has recently been formalized to allow quantification of direct and indirect effects in the presence of exposure-outcome interaction. More recently, these effects have been further subdivided into four separate component effects. We foresee that in coming years causal mediation analysis will become increasingly established in reporting of biomedical research. Of the four component effects, mediated interaction is considered intuitively most difficult to conceptualize and is usually thought of as blurring the separation of direct and indirect effects. In this paper, we demonstrated that mediated interaction might actually reflect established phenomena from clinical and biological sciences. We therefore suggest researchers using causal mediation analysis to realize that mediated interaction might actually indicate relevant processes in the pathways under study.
